# Bird Responses to Lowland Rainforest Conversion in Sumatran Smallholder Landscapes, Indonesia

**DOI:** 10.1371/journal.pone.0154876

**Published:** 2016-05-25

**Authors:** Walesa Edho Prabowo, Kevin Darras, Yann Clough, Manuel Toledo-Hernandez, Raphael Arlettaz, Yeni A. Mulyani, Teja Tscharntke

**Affiliations:** 1 Division of Conservation Biology, Institute of Ecology and Evolution, University of Bern, Bern, Switzerland; 2 Division of Agroecology, Department of Crop Sciences, Georg-August University of Göttingen, Göttingen, Germany; 3 Department of Forest Resources Conservation and Ecotourism, Faculty of Forestry, Bogor Agricultural University, Bogor, Indonesia; University of Fribourg, SWITZERLAND

## Abstract

Rapid land-use change in the tropics causes dramatic losses in biodiversity and associated functions. In Sumatra, Indonesia, lowland rainforest has mainly been transformed by smallholders into oil palm (*Elaeis guineensis*) and rubber (*Hevea brasiliensis*) monocultures, interspersed with jungle rubber (rubber agroforests) and a few forest remnants. In two regions of the Jambi province, we conducted point counts in 32 plots of four different land-use types (lowland rainforest, jungle rubber, rubber plantation and oil palm plantation) as well as in 16 nearby homegardens, representing a small-scale, traditional agricultural system. We analysed total bird abundance and bird abundance in feeding guilds, as well as species richness per point count visit, per plot, and per land-use system, to unveil the conservation importance and functional responses of birds in the different land-use types. In total, we identified 71 species from 24 families. Across the different land-use types, abundance did not significantly differ, but both species richness per visit and per plot were reduced in plantations. Feeding guild abundances between land-use types were variable, but homegardens were dominated by omnivores and granivores, and frugivorous birds were absent from monoculture rubber and oil palm. Jungle rubber played an important role in harbouring forest bird species and frugivores. Homegardens turned out to be of minor importance for conserving birds due to their low sizes, although collectively, they are used by many bird species. Changes in functional composition with land-use conversion may affect important ecosystem functions such as biological pest control, pollination, and seed dispersal. In conclusion, maintaining forest cover, including degraded forest and jungle rubber, is of utmost importance to the conservation of functional and taxonomic bird diversity.

## Introduction

Land-use change is inevitable: the growing human population, increasing wealth, and development of global markets lead to increasing demands for natural products, and as a response, natural ecosystems continue to be converted to agriculture [1]. This expansion endangers wildlife biodiversity as well as the capacity of the ecosystem to continue delivering services [2]. The quest for more cropland has put more pressure on tropical regions, which often offer lower production costs and less environmental regulation [3]. Tropical lowland forest is one of the most biodiversity-rich terrestrial ecosystems, but with the continuing expansion of agricultural land, that biodiversity is in a fragile state, in particular in Southeast Asia [4].

The island of Sumatra has experienced rapid land-use change. Indonesia’s annual forest cover loss is now estimated to be the highest in the world [5], and by 2010 70% of Sumatra’s forested area had been converted [6]. In Jambi, rubber agroforestry and oil palm agroforestry respectively covered 1 284 003 and 941 565 ha, summing up to 45% of anthropogenic land-use cover [7]. Plans by the Indonesian government to double its palm oil production by 2020 suggest this trend will continue [8]. In the face of dwindling forest cover, the question arises as how to compensate for the natural habitat losses and their impact on functional and taxonomic biodiversity.

In Southeast Asia, the response of bird communities to forest conversion has been documented before. In Thailand, bird diversity is lower in commercial rubber and oil palm plantations than in forest, and frugivorous-nectarivorous, insectivorous, and forest species are most affected [9]. Similar findings were reported by Thiollay for agro-forests (including rubber) situated in separate regions of Sumatra [10]. While monoculture agro-forests are also omni-present in our study region, agricultural systems with a more complex structure and plant composition might serve as refuges for plants and animals. Two such land-use systems are commonly found in our study region in the form of jungle rubber—a forest-like rubber plantation—and homegardens–defined here as a multistory combination of trees and crops around the homestead [11]. Despite that, jungle rubber is not equivalent to forest in conservation terms, but it can harbor more birds than rubber monocultures [12]. Homegardens are another diverse land-use system but on a smaller scale, and they are widely adopted by Indonesian households as they can support households in times of food shortage or generate additional income [13]. They are traditional agricultural systems characterized by low intensity management and high plant biodiversity, sometimes resembling natural forest [14]. However plant species richness and structure are variable and determined by social and geographic factors [15]. Jungle rubber and homegardens are traditional agricultural systems with potential for conservation due to their high plant species richness and structural heterogeneity. Jungle rubber however may be threatened, as it was once prevalent in the province of Jambi but it is being replaced by monospecific plantations [16].

The effect of land-use change on birds in the relatively small-scale mosaics of smallholder rubber plantations, oil palm plantations, jungle rubber, remnant forest blocks and homegardens, have never been jointly investigated. Existing studies sampling birds in rubber habitats usually come either from large-scale commercial plantations [9], or from older, intensive single-site transect studies on a subset of these systems [12]. Oil palm plantations have been more intensively studied by Azhar et al. in peninsular Malaysia [17], [18], but without comparison to other agricultural land-use systems. In Indonesia, smallholder oil palm plantations cover an equally large area as commercial plantations [19] but they have lower intensity management and yields, thus having greater potential for biodiversity conservation [20], [18], although some evidence suggests that the difference may be negligible [21]. The bulk of the rubber production in Indonesia is also coming from smallholder plantations [22] with low management intensity. Valorising these small-scale agricultural habitats could not only help to meet conservation targets by maintaining a more heterogeneous landscape for bird communities; it could also enhance their own ecological functions, since birds can control arthropods pests [23], disperse seeds, and pollinate plants [24].

Here we use lowland rainforest as a reference to investigate the effect of conversion to different land-use types on the total bird abundance, the abundance within feeding guilds, different measures of species richness, and species composition of bird communities, and evaluate the conservation value of these habitats. We conducted bird point counts in 32 plots of 4 different systems: lowland rainforest, jungle rubber, rubber plantations and oil palm plantations, which are the main land-use systems in the landscape mosaic in Jambi. We additionally sampled birds in 16 homegardens that represent small-scale traditional systems, situated in the same study region to evaluate their contribution to conservation. We expect that bird species richness is highest in forest due to the more diverse food resources and structural complexity, and lowest in the monocultures. Homegardens and jungle rubber may have intermediary biodiversity levels and thus act as refuges for forest species because of their high plant species diversity and structural heterogeneity. We also expect that some groups, such as feeding generalists (omnivores), reach higher abundances in the monocultures because these species are more flexible in their diets and can thus take advantage of disturbed systems’ food resources.

## Materials and Methods

Data are publicly available on Dryad (doi:10.5061/dryad.g77m8). The study plots are privately owned except for the forest sites, which are situated in the protected forests Harapan Rainforest (PT. Restorasi Ekosistem Indonesia) and the Bukit Duabelas National Park.

Bird field sampling was observational and carried out by the first author, who is Indonesian, so no research or collection permit was required. The joint co-authors' research permit (number 211/SIP/FRP/SM/VI/2012) was recommended by the Indonesian Institute of Sciences (LIPI) and issued by the Ministry of Forestry.

This research involved human participants from 16 households for the homegarden survey. No institutional review board or ethics committee was available in our project region. Instead, the survey was approved by the Unit Management office of the CRC 990 at the local University of Jambi and by the project's Indonesian counterparts. Local authorities (village leaders) were informed of the survey with official letters from the Unit Management office. Complying with Indonesian customs, simple verbal consent was obtained from each household head for sampling in their homegardens and participating in the interview. Participants signed receipts for obtaining monetary compensation after the interview. Personal data were first recorded in the field for identification purposes and later removed for the statistical analysis and deposition on Dryad.

### Study sites

**Fig 1:**
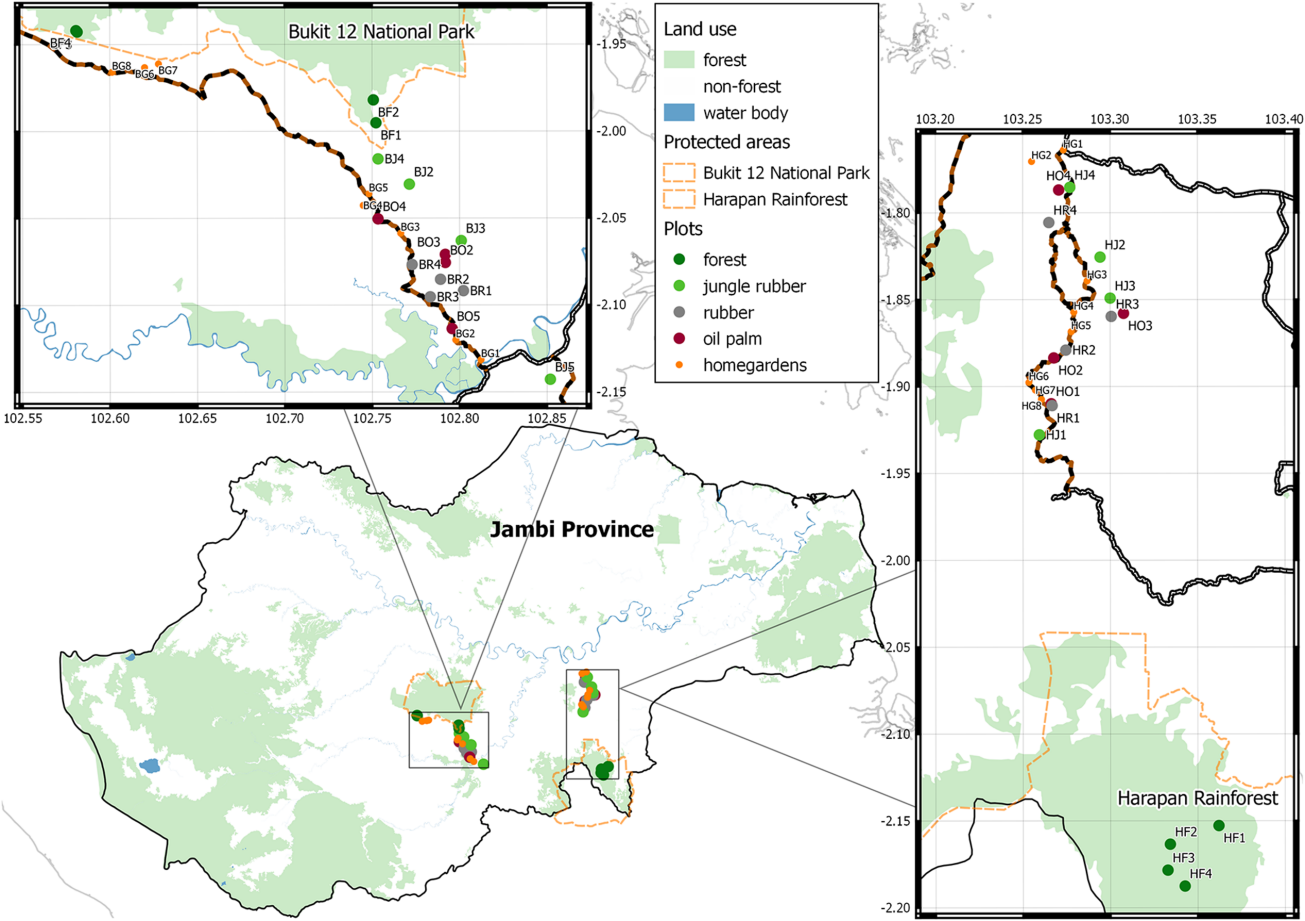
Map of the plots and homegardens in Jambi Province, Sumatra, Indonesia. The first letter of plot and homegarden codes indicates the region (H: Harapan, B: Bukit Duabelas), the second letter the land-use type (F: forest, J: jungle rubber, R: rubber, O: oil palm, G: homegarden). Forest cover is derived from Landsat 2013 (data from USGS/NASA Landsat, imagery interpretation courtesy of Dian Melati).

The study sites are situated near two protected forests in Jambi Province, Sumatra, Indonesia (Fig 1). Thirty-two plots, 50×50m in size, with 16 plots per region, were established on lowland rainforest and 3 transformed habitats: jungle rubber, rubber (*Hevea brasiliensis*) plantation and oil palm (*Elaeis guineensis*) plantation in the frame of the CRC 990 (EFForTS) project. Our lowland rainforest sites are natural forests that have experienced some disturbance such as clearing and logging. Jungle rubber is planted rubber with secondary forest re-growth and minimum management practices [16]. Rubber plantations and oil palm plantations are intensively managed monoculture plantations covering an extensive area of the province. The study design and region is described in detail by Drescher at al. [25]. The mean pair-wise distance between sites was 11.5 km and 18.2 km for the Bukit Duabelas and Harapan regions, respectively.

We additionally selected 8 homegardens, which were not part of the core study sites of the CRC 990, in each region. Homegardens were separated by a minimum distance of 100 m and it was not possible to have larger separation between them since they are invariably tied to human settlements along roads. The selected homegarden areas ranged between between 100 to 300 m^2^, smaller or larger homegardens (observed range 50–800m^2^) were either too small for meaningful bird surveys, or, for the larger ones, too rare and unrepresentative to include in our sample. The typically small size of homegardens compared to plots prompted us to double the amount of homegardens (16 homegardens vs. 8 plots per land-use type).

### Bird survey

We conducted point count surveys in the centre of the plots in forest, jungle rubber, and rubber and oil palm plantations. Each plot was visited four times, except five plots (BF1, BF2, BJ2, BJ4, and BO1, see Fig 1) which could only be visited three times due to time constraints. Each homegarden was also visited four times, except two homegardens (BG1 and BG2) which could only be visited three times due to time constraints. Each point count visit lasted 20 minutes. In the homegardens, birds were observed from the best possible vantage point. Individuals flying through or above the plot or homegarden were excluded. Each site was surveyed from June until July 2013, during non-rainy days. Cloud cover during the visit was estimated by eye and expressed in percent as clouded weather can possibly affect bird activity [26].

Birds were surveyed by sound and sight between 6:00 and 10:00 by the first author using 7×42mm binoculars (Nikon Monarch). Species identity, number of individuals, used vegetation layer (only for plots: ground, understory, middlestory, canopy, emergent trees) or used plant species (only for homegardens), horizontal distance from the observer (measured by a digital rangefinder Nikon Laser 1000AS) were recorded. The timing of bird data collection randomly alternated between early to late morning to minimize a bias due to the observation time [27, 28]. Unfamiliar bird calls were recorded using a directional microphone (Sennheiser ME-66/K6) coupled to a digital sound recorder (Olympus LS-3). The recordings were later compared with online databases: the public bird call database Xeno Canto (www.xeno-canto.org) and our own database SoundEFForTS (http://soundefforts.uni-goettingen.de/). Bird species identification in the field followed Mackinnon, Phillips & van Balen [29], but we used Birdlife international [30] taxonomy in the analysis.

### Homegarden survey

Semi-structured interviews (S1 Questionnaire) with the homegarden owners showed they were tended by people aged 21 to 71 years, and mostly by women or both genders (13 out of 16 homegardens). Homegarden products were used for personal consumption and only occasionally sold (3 households). Fertilizer and herbicides were used in 13 homegardens, with insecticides used in 8 homegardens. On average, the ground cover consisted of 65% of vegetation and litter, while the rest was mostly bare ground. We could identify a total of 109 cultivated plant species, of which 42 were tree species. An average of 18 (sd: ± 7) identified cultivated plant species per homegarden was found, of which an average of 7 (sd: ± 4) species were trees. The most common plants (found in more than half of the plots) were chili pepper (*Capsicum frutescens*), papaya (*Carica papaya*), coconut trees (*Cocos nucifera*), turmeric (*Curcuma longa*), lemongrass (*Cymbopogon sp*.), sweet potato (*Ipomea batatas*), mango (*Mangifera indica*), cassava (*Manihot esculenta*), banana (*Musa sp*.), sugarcane (*Saccarum officinarum*), katuk (*Sauropus androgynus*), and ginger (*Zingiber sp*.). Generally, homegardens had few tall trees and they were never integrated in remnant forest habitat (for an example see S1 Fig),

Due to their inevitably smaller areas (mean ± sd = 220 ± 51 m^2^), the 16 homegardens’ bird abundance, richness, feeding guilds, and communities were not comparable statistically to those from the plots (area: 2500 m^2^). In one homegarden (HG8), the owner “cleaned” all vegetation so that the bird survey could not be conducted.

### Data analysis

#### Diet, stratum, habitat and Red List status

All birds were classified into feeding guilds (insectivores, frugivores, granivores, omnivores, and nectarivores) based on their primary diet. The classification is mainly based on Thiollay [10] and was completed with data from the Handbook of the Birds of the World [31]. We used the same sources, as well as Beukema et al. [12], to complement the data with the birds’ preferred habitat use as defined by Thiollay [10] (primary and old secondary forest interior; forest gaps, edges, or upper canopy; little wooded and cultivated areas). Data about the foraging strata were extracted from Wilman et al. [32] (tree crown; bark and wood at any height; understory, mostly foliage and epiphyte; grass shrubs in open areas). We plotted the abundance per feeding guild, habitat type, or foraging strata in each land-use type and region and only used the data from the first three visits to avoid a bias due to several sites missing the fourth visit. We supplemented the bird data with information from the IUCN Red List [33] to show the number of species of conservation importance.

#### Response variables

We used the sum of the maximum of simultaneously detected individuals per species as a conservative measure of abundance. Abundance was computed per point count visit to be comparable with our measure of species richness per point count visit. We also analysed the abundance in each feeding guild and the rarefied richness per plot after 3 visits, and the total richness per land-use type after 3 visits was visualised. For the per-visit richness and abundance, we also counted birds that were only identified to genus level if they differed from the genera of the other observed birds during the same visit.

For homegardens, we computed the average number of species and abundance of birds, counted after three visits. We also pooled data from all 16 homegardens, obtaining a total area of 3297 m^2^, which is more similar to the area covered by our point counts in core plots, to count the total species richness.

#### Statistical models

All analyses were carried out using R software version 3.2.1 [34] and graphs were made using the ggplot2 R package [35].

For all per-visit count data (abundance and richness), we fitted generalized linear mixed-effects models (GLMER, glmer function from R package lme4) with plot as a random effect and Poisson family. Rarefied species richness yielded one data point per plot and as a consequence, it was modelled using a generalized linear model (GLM) with Poisson family (without random effects). For all models, we started with the full set of explanatory variables, including land-use type and region (categorical variables) as well as their interaction, and scaled cloud cover (z-transformed to avoid convergence issues in GLMERs). The full model’s overdispersion, homoscedasticity and residual normality assumptions, as well as outliers, were checked using diagnostic plots. We generated all possible models based on all combinations of predictors included in the full model and ranked them by AICc [36] (dredge function from R package MuMIn). All variables that were included in the best models (models within 2 ΔAICc scores from the best model) were used to construct a model which served in a subsequent post-hoc pairwise comparison of land-use types with forest (glht function from R package multcomp [37]), and we report the results whenever a global Chi-square-test was significant (P<0.05). Total richness per land-use type was not analysed statistically since it is an aggregated measure.

#### Community composition

We visualized the bird community composition using non-metric multidimensional scaling (NMDS) based on Bray-Curtis distances derived from an abundance community matrix (R package vegan [38]). We performed permutational multivariate analysis of variance tests (adonis function from R package vegan) to detect the difference of each transformed land-use’s community with forest.

## Results

Based on our point counts, we detected 451 birds representing 71 species and 24 families, as well as 74 birds from 13 species and 7 families, respectively in core plots and in homegardens. Sampling intensity was comparable between land-use types, and rarefaction analysis supports our finding (S2 Fig), although forest appears to have been under-sampled. Among all detections, 25 were only identified to genus level and 9 detections remained wholly unidentified. Twenty genera were only found in forest, 4 only in jungle rubber, 6 only in rubber, and 5 only in oil palm. Birds were usually recorded more often close to and also far away from the observer in the plantations (S3 Fig). The estimated cloud cover was highest in forest and lowest in rubber (S4 Fig), but we did not test for statistical significance.

### Total bird abundance and species richness

**Fig 2:**
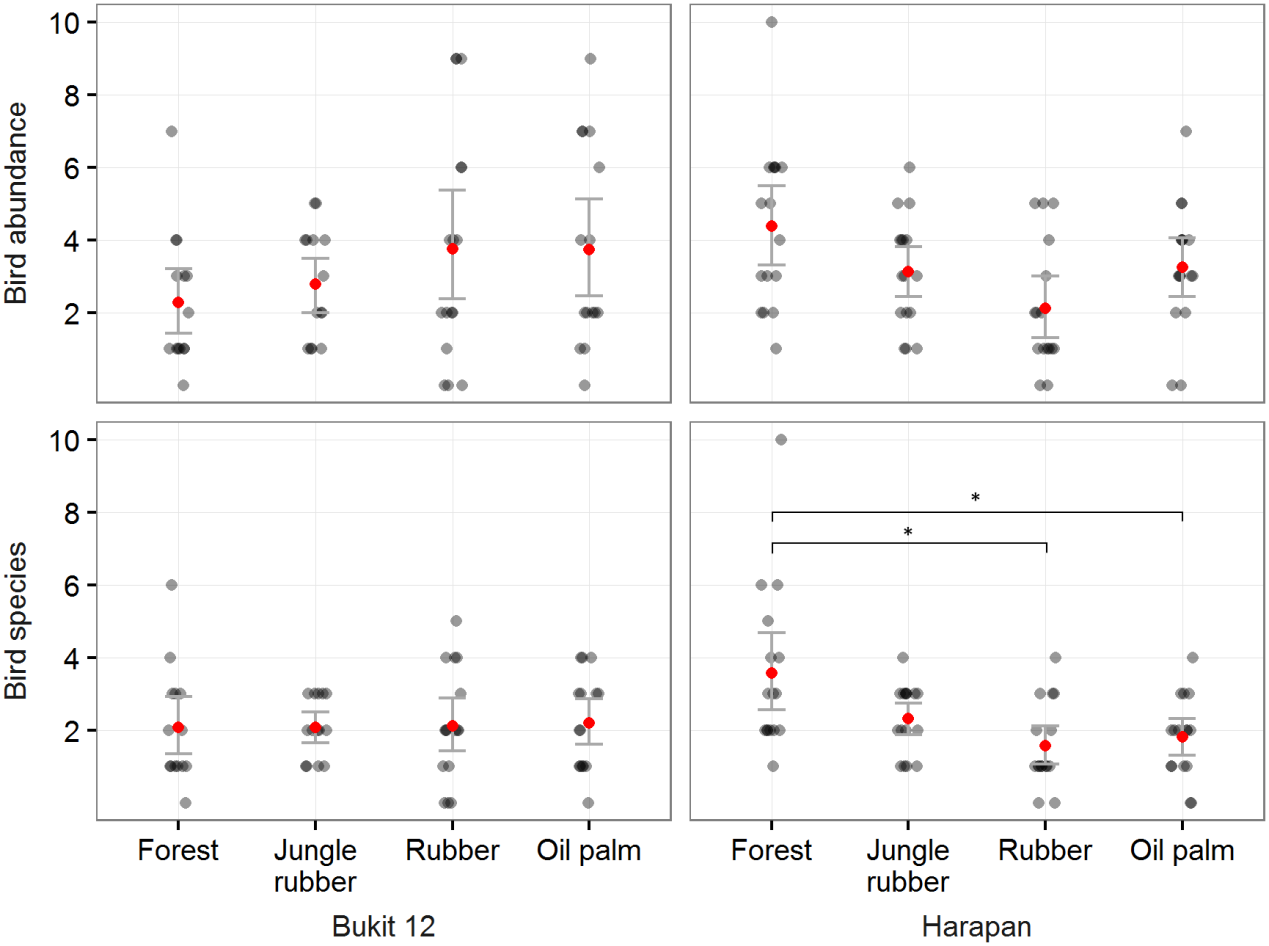
Bird abundance and species richness per 20 minute visit, split up by land-use type for two regions in the province of Jambi, Sumatra. Black dots represent visits, red dots represent mean values per land-use type, error bars represent the mean standard error, asterisks denote statistical significance in post-hoc multiple comparisons with forest.

**Fig 3:**
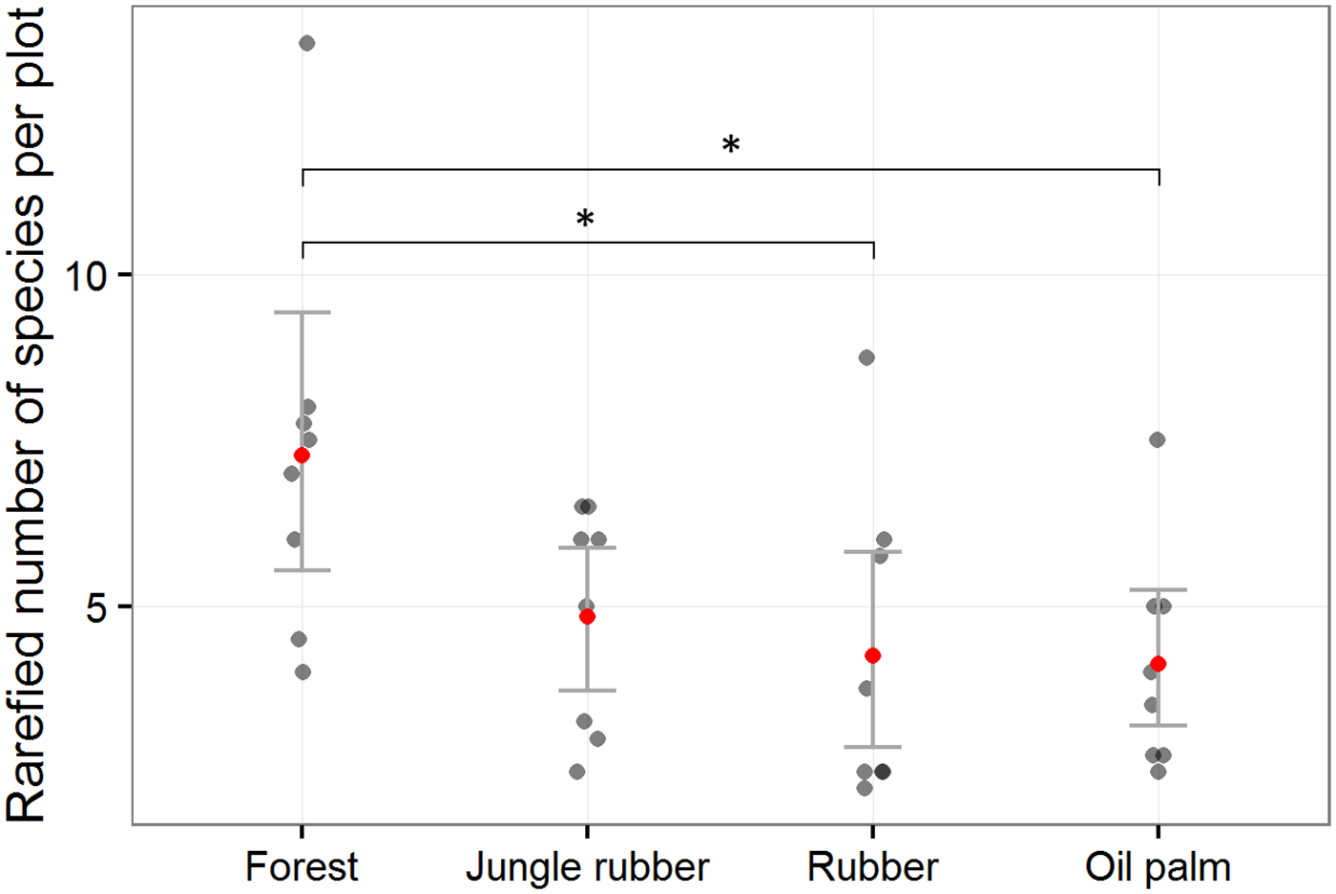
Rarefied species richness after three 20 minute visits, split up by land-use type for both regions combined in the province of Jambi, Sumatra. Grey dots represent plots, red dots represent mean values per land-use type, error bars represent the mean standard error, asterisks denote statistical significance in post-hoc multiple comparisons with forest.

The best model for bird abundance per visit was the null model, and there were no other models within 2 ΔAICc, so no global test was needed. The bird abundance per visit followed different trends among regions (Fig 2). The best models (within 2 ΔAICc) for bird abundance per feeding guild (using only insectivores and omnivores for which we had enough detections) did not contain land-use type or region as predictors, so no global test for assessing differences between land-use type and region was conducted. The best models for species richness per visit contained land-use type and region predictors. The global Chi-square test for the contrasts between land-use types was significant (P<0.05) and the multiple comparison showed significantly (P<0.05) higher values in forest than in the rubber and oil palm plantations for the Harapan region. The best model for rarefied species richness contained only land-use type as a predictor: there were no other models within 2 ΔAICc. The global test for the contrasts between land-use types was significant (P = 0.01) and showed significantly (P<0.05) higher values in forest than in the rubber and oil palm plantations (Fig 3). For all responses, cloud cover was dropped from the predictors as it was never contained in the best models within 2 ΔAIC.

**Fig 4:**
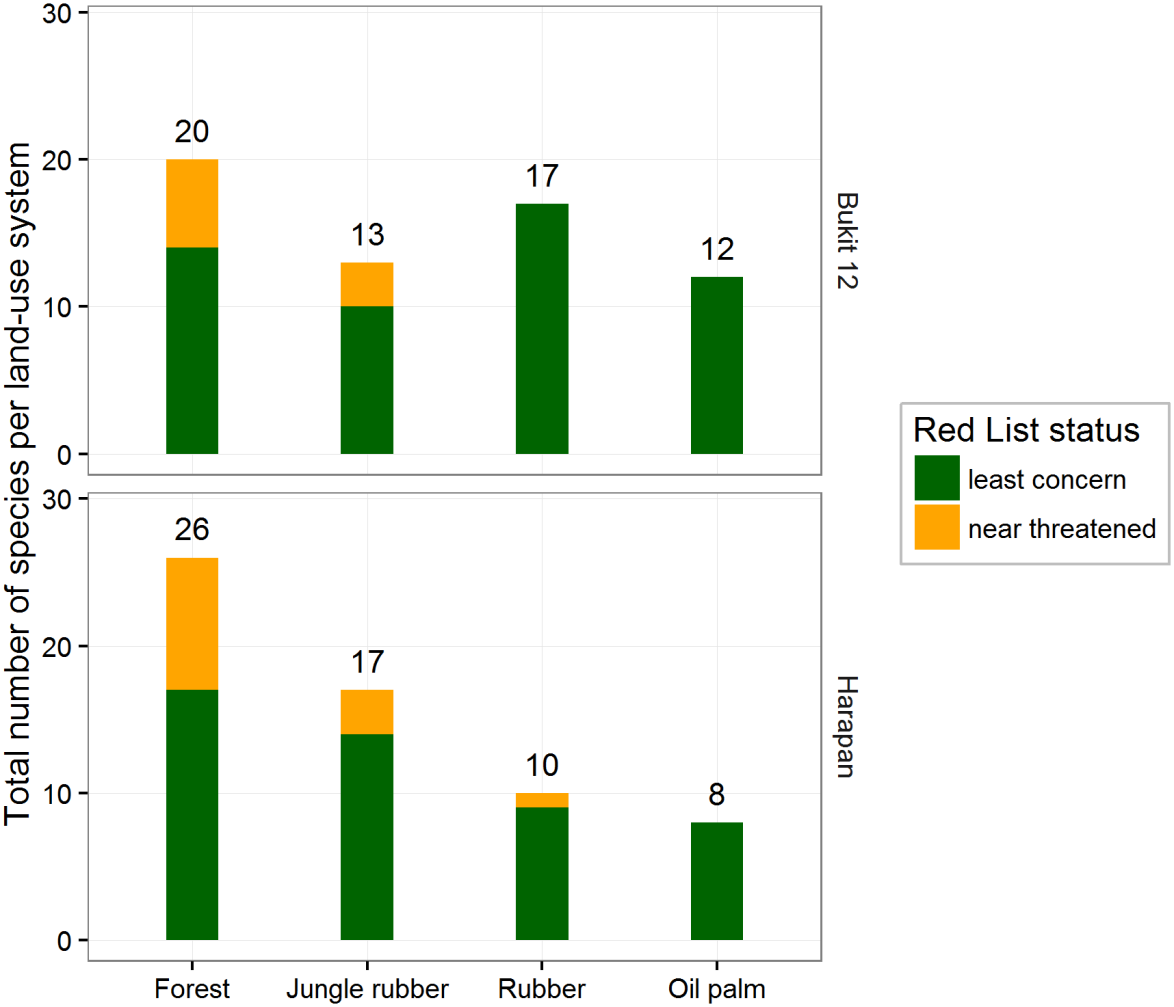
Total bird richness after three 20 minute visits in each land-use type and IUCN Red List threat status for two regions in the province of Jambi, Sumatra. For some unidentified birds, the threat status was not available (NA).

In both regions, the total species richness per land-use type was highest in forest (see Fig 4). The species richness in rubber sites of Bukit Duabelas was relatively high, even surpassing the species richness in jungle rubber. In both regions, species of conservation concern (“near threatened” category according to IUCN Red List) were increasingly rare along the land-use conversion gradient and mostly absent in plantations.

In 4 of the 15 homegardens, we did not find any bird even after four visits. After 3 visits, the average total species richness per homegarden was 1.6 (sd: ±1.4) with a maximum of 5 species, and the mean number of detections per visit was of 1.15 individuals. All 15 homegardens pooled together had a species count of 11 after three visits.

### Feeding guild abundances

**Fig 5:**
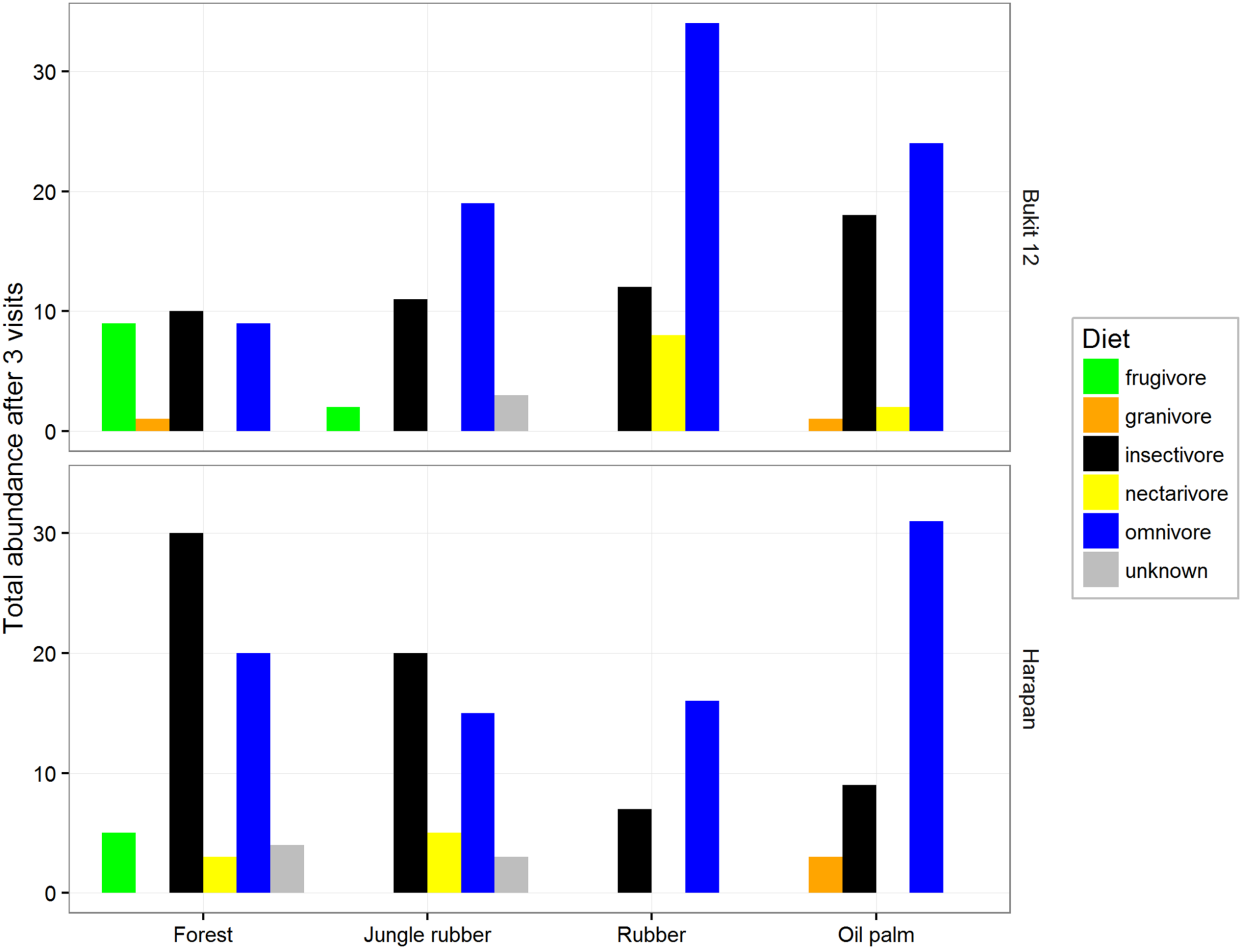
Bird abundance after three 20 minute visits in each feeding guild per land-use type for the two different regions of the province of Jambi, Sumatra. Feeding guild categories were based on Thiollay et al. (1995).

Insectivores declined in converted land-use types in the Harapan region, and the trend was reversed in the Bukit Duabelas region (Fig 5); the highest omnivore abundances were observed in plantations, but both trends were not significant (global Chi-square test P = 0.18), so we refrain from a more detailed description of these results. Frugivores were absent from plantations in both regions, while nectarivores were only present in plantations at Bukit Duabelas, and in forest and jungle rubber at Harapan. Granivores made up a minor proportion of detections and were mostly found in oil palm.

In homegardens, we predominantly found granivores (22 individuals) and omnivores (17 individuals). Nectarivores were still common (10 individuals), while insectivores were rare (3 individuals).

### Community composition

**Fig 6:**
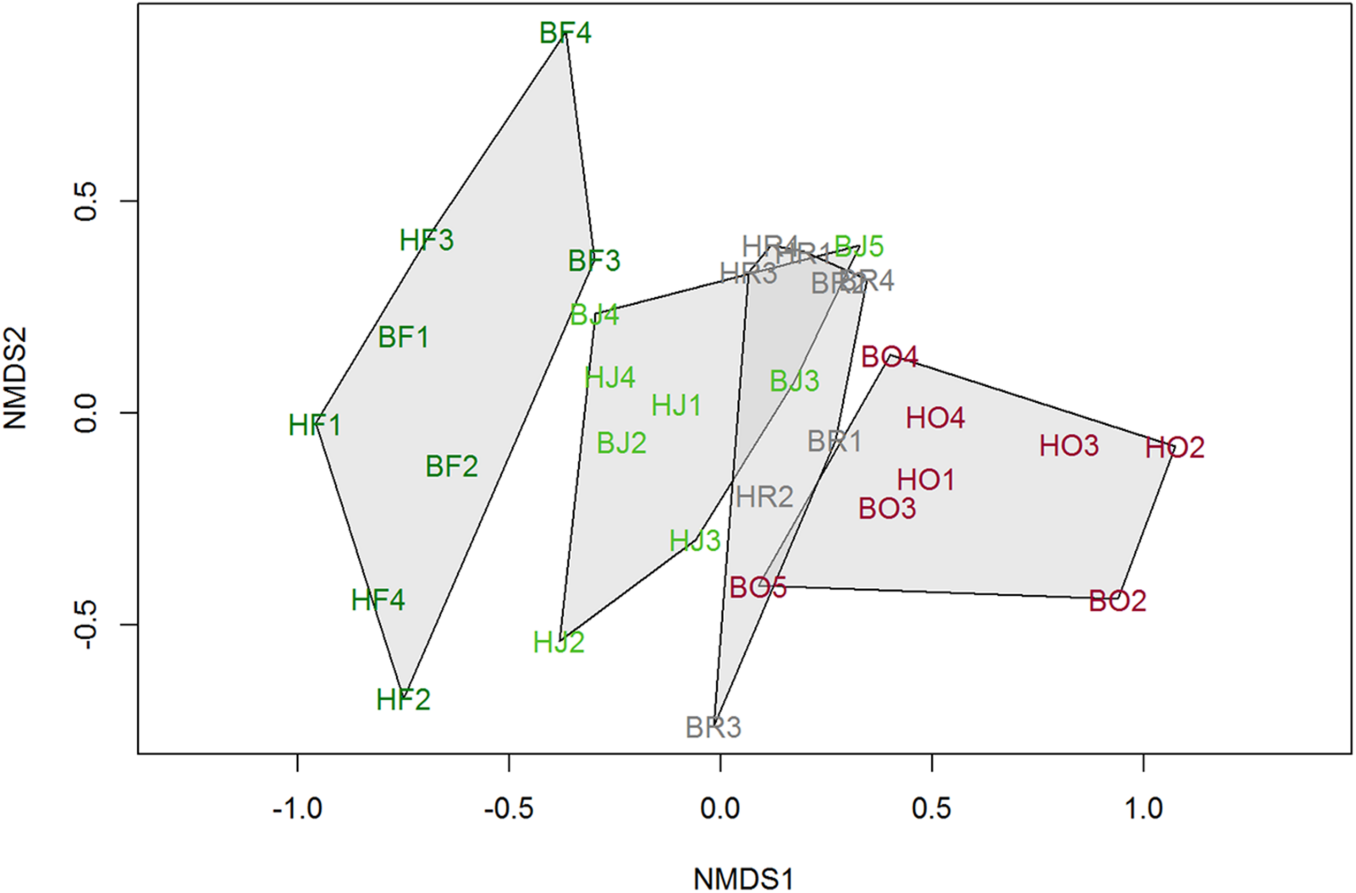
Non-metric Multidimensional Scaling of bird communities in different land-use types of Jambi, Sumatra. Graph based on abundance data from both regions.

Two dimensional ordination visualization of bird communities based on abundance data showed that the forest community was different from the other communities in the converted land-use systems (Fig 6). Clear overlap was visible between jungle rubber and rubber plantations, and to a lesser extent between rubber and oil palm plantations. The ADONIS analyses revealed that land-use type was significant in partitioning the bird communities, and that forest communities were significantly different from each of the other communities in converted land-use types (all p<0.001).

## Discussion

We found no differences in terms of abundance between the land-use types, but both richness per visit and rarefied richness per plot were significantly lower in plantations compared with forest. Frugivores were absent from monocultures; the trends in abundance of insectivores and omnivores are statistically not significant, so we do not interpret them further. Richness per land-use type decreased along the transformation gradient, with almost no bird species of conservation concern present in monocultures. Bird communities in jungle rubber, rubber and oil palm monocultures were significantly different from forest communities.

### Diversity along the land-use conversion gradient

Overall, species richness decreased from forest to plantations. In our region, forest harboured more than twice the number of species compared to oil palm plantations, and many more genera were restricted to forest habitats than to plantations. However, the species richness decline was not as sharp as the one observed by Aratrakorn et al. in commercial plantations [9], where losses of at least 60% bird species were reported: we documented losses of 43–45% in rarefied richness in plantations compared to forest. It suggests that smallholder agricultural plantations might be less detrimental for birds, due to their inherently smaller sizes contributing to a higher diversity of habitats in the landscape mosaic. Species groups that went missing after conversion of forest are hornbills, trogons, barbets, woodpeckers, flycatchers, and some babblers. The lack of large canopy trees (for hornbills), standing dead trees (for woodpeckers), woody understory growth (for babblers and understory foragers), might have greatly affected these forest species [10, 39].

We expected that monoculture rubber would be more hospitable to birds than oil palm plantations: the branched rubber tree structure may provide more space for foraging, perching, or nesting. Even though rubber is harvested more frequently, tapping takes place at the trunk base, while harvesting in oil palm occurs in the middle of the canopy, which is potentially more disturbing. Although several bird species are able to shift their foraging height in response to disturbance [40], oil palm plantations clearly cannot harbour tree crown species, integrating open-land species instead (S5 Fig). However all in all, we could not detect differences in abundance or richness between the rubber and oil palm plantations, and their communities were relatively similar (Fig 6), which was also found by Aratrakorn et al. [9]. Both plantation types are novel, simplified monocultures with a high disturbance regime due to human management activities, explaining the high prevalence of similar generalist species in both systems.

### Differences in feeding guild responses

In our study, frugivores were seldom found in jungle rubber compared to forest, and were entirely absent in the monoculture plantations. Therefore, we deduce that bird seed dispersal may be severely decreased in transformed systems. This result was consistent with other findings in Sumatran agroforest [10], in Hainan rubber plantations [41], and in Thai commercial rubber and oil palm plantations [9]. Availability of fruits, which fluctuates strongly between seasons and years even in natural forests [42], is crucial for maintaining frugivores, and the presence of fruiting remnant forest trees in jungle rubber could make a decisive difference [43].

In Bukit Duabelas, we found nectarivores only in plantations, while they only occurred in jungle rubber and forest in Harapan. Although agroforestry systems, when adjacent to open areas, may result in spillover of nectarivore species [44], we suggest this increase is mainly due to the *Hevea* blooming during the bird survey in Bukit 12. Rubber provides large extra-floral nectar resources during the blooming period, and Li et al. [41] suggest that birds utilize them, as they also observed similar temporal trend of nectarivores. We hypothesize that nectarivorous sunbirds (which are facultative insectivores) might also be attracted by the insect pollinators of rubber (thrips and midges, pers. obs. YC). Flowerpeckers (*Dicaeum trigonostigma*) might use rubber as well, as they were reported to consume rubber flowers directly [45]. Bird pollination thus seems to be decreased in plantations and only temporarily upheld in rubber plantations during the blooming time, when bird pollinators are attracted.

Especially in Harapan, insectivore abundances decreased from forest to jungle rubber and then to rubber. In Bukit 12 however, insectivore abundance increased in plantations. Almost all insectivores found in oil palm are open land and common species such as *Prinia* and *Orthotomus*. Forest-dependent insectivores, such as Grey-chested Jungle-flycatcher *Rhinomyias umbratilis*, Scarlet-rumped Trogon *Harpactes duvaucelli*, and Banded broadbill *Eurylaimus javanicus* were never found outside the forest sites. At the same time, a large portion of understory insectivores, such as babblers, were found almost exclusively in forest and jungle rubber, as they are sensitive to habitat degradation [46]. They could not be found entering deep into the plantations, even when observed in nearby jungle rubber fragments (pers. obs. WEP). The absence of undergrowth vegetation on plantation plots seems to be affecting this group greatly [9]. Bark gleaning insectivores like woodpeckers and nuthatch were entirely missing in oil palm. Under these circumstances, we are unsure whether pest predation services–which might be relevant for oil palms [47]–are hampered or enhanced in plantations. There are indications that common species (like *Pycnonotus*, *Orthotomus* and *Prinia* species) comsume a broad variety of insects in oil palm, but not specifically pests [48]. Consequently, biocontrol effects could be positive or negative, as birds may affect pests as well as their natural enemies. Note that overall, the diversity of the insectivorous feeding guild is strongly reduced (S6 Fig, 16 species in plantations combined versus 29 in forest and jungle rubber combined).

In rubber and oil palm plantations, we generally observed similar species compositions with omnivores such as *Pycnonotus goiavier* and *Dicaeum trigonostigma* dominating. Overall, omnivores were more than twice as abundant in the plantations compared to the forest, but they had only slightly lower observed richness (13 species in plantations combined versus 16 in forest and jungle rubber combined). Forest omnivores such as leafbirds, fulvettas, and scimitar-babblers, were replaced by other species that have adapted well to anthropogenic habitats, such as bulbuls (*Pycnonotus spp*.). In summary, our results show that there is a feeding guild composition change towards less specialized birds in the simpler habitat types such as plantations [43, 44, 49]. It is uncertain whether the omnivorous birds can still fulfil the seed dispersal function of the missing frugivores in plantations, as only dietary analyses could reveal that.

### Confounding factors and recommendations

Differences in bird detectability between land-use types should hardly bias our results since we only recorded birds that are within the plot (maximum distance 35m). However, we found that birds in forest and jungle rubber were rarely detected in the close vicinity of the observer, suggesting in these natural systems birds are usually more secretive. We could not correct for this minor bias so our estimates are conservative for forest sites. Moreover, the weather conditions were usually worse in forest than in the other systems, while the skies were clear during the rubber plot visits. While we could account for that in our models, the weather covariate was always dropped, suggesting that it may not affect our outcomes significantly (see also [50]).

Both regions showed strikingly different trends in the abundance, species richness and number of conservation-relevant species for forests and rubber plantations, suggesting that regional effects are of major importance. While we cannot test or quantify the regional effect with only two studied regions, we know that especially the forests were dissimilar: in Bukit Duabelas, law enforcement was ineffective as bird trapping and rubber tapping were still ongoing in the vicinity of our plots. The Harapan forest sites were better protected and could also benefit from ongoing restoration efforts.

Other factors may influence the observed abundance and richness trends. Indeed several studies [9, 17, 21] showed that understory presence could positively affect bird species richness. We did not test for such an effect, but our highest abundance and richness values were in plots with dense and tall undergrowth (pers. obs. WEP). The importance of rubber blooming was mentioned above and indeed, we observed higher nectarivore abundances in the Bukit 12 survey when rubber was blooming. Another possible influencing factor is the distance from the forest fragments. Aratrakorn et al. [9] did not find any evidence for this in their commercial plantations, but Azhar et al. found effects for arboreal omnivores and terrestrial frugivores [20]. In our Bukit 12 region however, plots from converted land-uses were closer to the forest border than in Harapan (on average 3.2 km versus 6.0 km for Harapan, Fig 1). We suggest that the proximity to forest could explain the relatively high species richness and abundances in the Bukit 12 converted land-use plots. All things considered, we recommend that during bird surveys in rubber plantations, blooming events should be noted, and in plantations, understory density should be quantified. A designed gradient in distance from the transformed plots to the forest (as a reference system), combined with a more sophisticated land-use cover analysis, could impart the influence of forest proximity on bird communities, which should ideally be tested in different regions.

### Conservation implications

Despite being perennial habitats, monospecific plantations are too simplified to harbour as many species as forests. Our results show that among the transformed habitats on the study site, jungle rubber still plays an important role in harbouring bird species, particularly forest species that cannot survive in structurally much simpler habitat (S7 Fig): jungle rubber in both regions harboured 14 forest species, which was more than twice the number in both monoculture plantations combined (6 species). Strikingly, the entire feeding guild of frugivores was missing from the moncultures. Jungle rubber communities were intermediate in composition between forest and plantation communities (Fig 6), and almost half of the 15 near threatened species from the forest were found in jungle rubber. As a buffer, jungle rubber favours forest bird species due to its vegetation structural complexity and plant diversity (including fruiting trees), as it resembles secondary forest more than the other types of land-use [51].

Despite encouraging evidence from other parts of the world that homegardens can sustain bird communities and thus support conservation targets [52], we found that single homegardens in our region were quite irrelevant for conserving bird communities. The gardens were irregular in shape, leading to possibly stronger edge effects, markedly smaller in size than the study plots, and located near to roads and households, which are a source of disturbance. Homegardens may be attractive to birds for its food-resources. Therefore, owners may perceive them as a threaten to the crops, and in some cases use visual methods such as birds-scarers to dissuade birds from entering into the homegardens (pers. obs. MTH). Additionally, we found a low plant species richness and structure and full-grown trees were rare (pers. obs. MTH). In contrast, homegarden studies in Sulawesi and Java respectively recorded from 28 to 37 and from 42 to 58 plant species per homegarden [53, 54]. In contrast however, when pooling homegarden data for a fairer comparison of species richness with core plots at similar areas, we found a high number of species (11). Although this number is inflated due to the geographical separation between homegardens (high beta-diversity) it comes second after the highest species richness recorded in plots. Therefore, although homegardens certainly do not provide breeding habitat for birds due to their high disturbance regime, they can apparently provide resources that are used by many bird species.

Bird communities can be preserved with several management practices. Favouring a more diverse tree structure and habitat in plantations can be achieved by planting trees in cohorts of varying ages, with intercropping of rubber [55] and oil palm [56], keeping remnant forest trees–especially fruiting trees for the missing frugivorous trees [57]–and standing dead trees (“snags”) [58], and by maintaining a denser undergrowth [9, 17, 21]. Interestingly, some findings by Azhar et al. suggest that polyculture may not necessarily be beneficial for the overall bird species richness [59]. Finally at the regional scale, the matrix between the forested natural habitat and transformed habitat can be improved by keeping plantations small and forested systems connected [60].

Previously, the predicted land use development in Jambi incorporated a combination of jungle rubber along with monoculture plantations [16]. However recent changes show a shift to more profitable monoculture plantations[61]. Smallholders could maintain their jungle rubber agroforestry due to the minimal management costs and limited capital, but oil palm offers short term profits [16, 62]. The immediate challenge for conservation in the study area is the change of forest and jungle rubber towards a homogenized region dominated by large scale monoculture plantations.

Policies which support smallholders in maintaining the heterogeneity within the landscape mosaic are required. With the growing eco-sensitive market, smallholder rubber certification–despite its complexity–should be developed further [63]. A multi-stakeholder approach providing incentives for smallholders to maintain their jungle rubber is feasible as has already been implemented in the Bungo regency [64]. Favouring jungle rubber and mixed crop plantations, along with practices which maintain structural complexity of the plantation will benefit the environment and biodiversity more. Meanwhile, it is of utmost importance to protect and maintain the remaining forest cover.

## Supporting Information

S1 Fig An example of homegarden (BG1) sampled in our study. (JPG)

S2 Fig Rarefaction curves for each land-use type. Total species richness was rarefied to the lowest sample size occurring in jungle rubber, where 88 birds were observed. Total rarefied richness forest: 38; jungle rubber: 27; rubber: 25, oil palm: 16.
(TIF)

S3 Fig Histograms of bird detections in each land-use type depending on the distance from the observer, divided in bins of equal area. (TIF)

S4 Fig Estimated percent cloud cover during each core plot visit in each land-use type. Means are indicated in red, error bars represent the standard error of the mean.
(TIF)

S5 Fig Bird abundance after three 20 minute visits in each stratum preference group per land-use type for two regions of the province of Jambi, Sumatra. Strata preferences were obtained from Wilman et al. (2014).
(TIF)

S6 Fig Total bird species richness after three 20 minute visits in each feeding guild per land-use type. Feeding guild categories were based on Thiollay et al. (1995).
(TIF)

S7 Fig Bird abundance after three 20 minute visits in each habitat preference group per land-use type for two regions of the province of Jambi, Sumatra. Habitat preferences were mainly obtained from Thiollay et al. (1995).
(TIF)

S1 Questionnaire Homegarden household questionnaire. (DOCX)
