# Supplementary figures and images for: Bird Responses to Lowland Rainforest Conversion in Sumatran Smallholder Landscapes, Indonesia
Source: PLoS One. 2016 May 25;11(5):e0154876. doi: 10.1371/journal.pone.0154876 (PMC4880215; doi:10.1371/journal.pone.0154876)

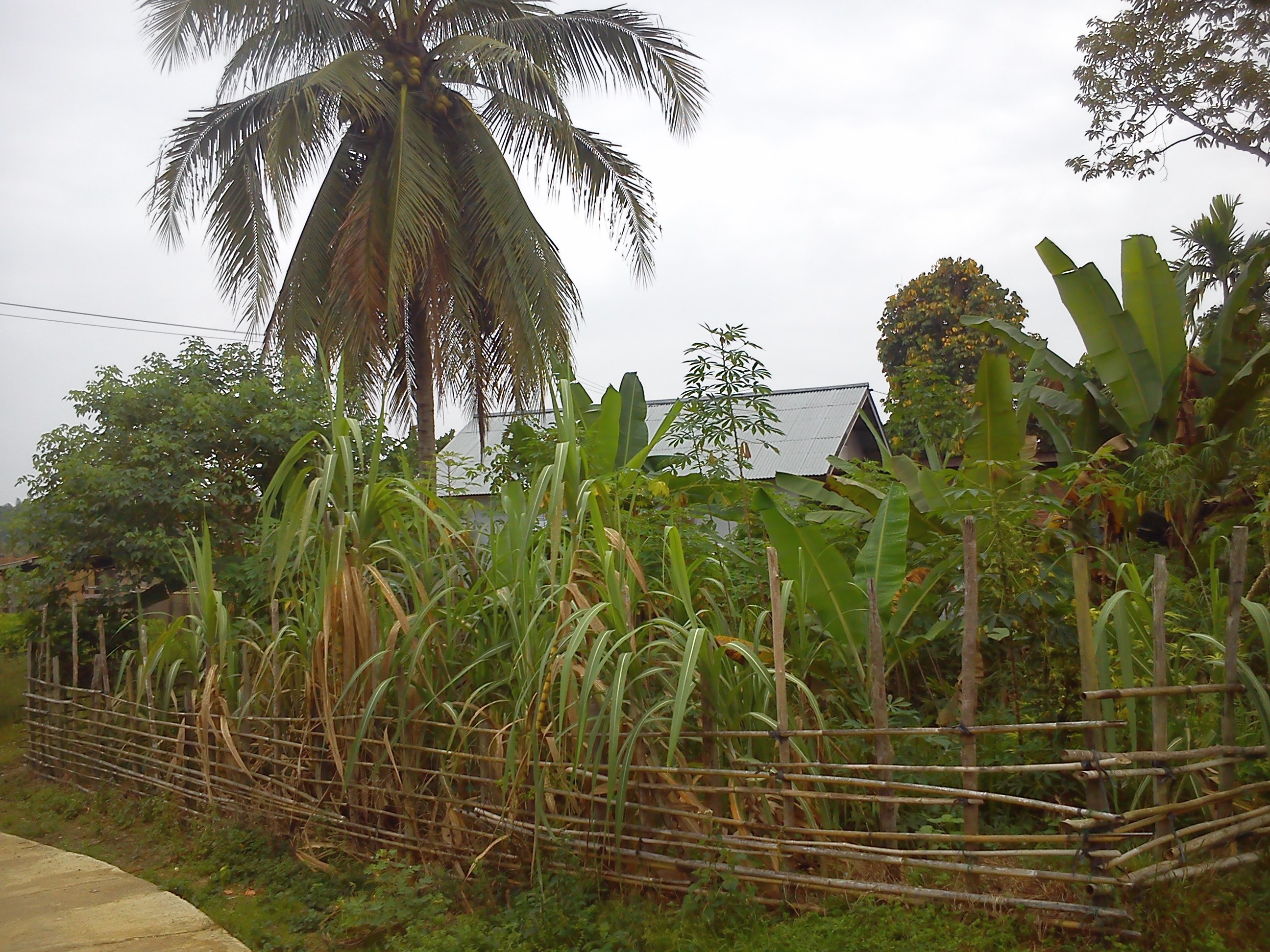

Supplement: S1 Fig — (JPG) [file pone.0154876.s001.jpg]

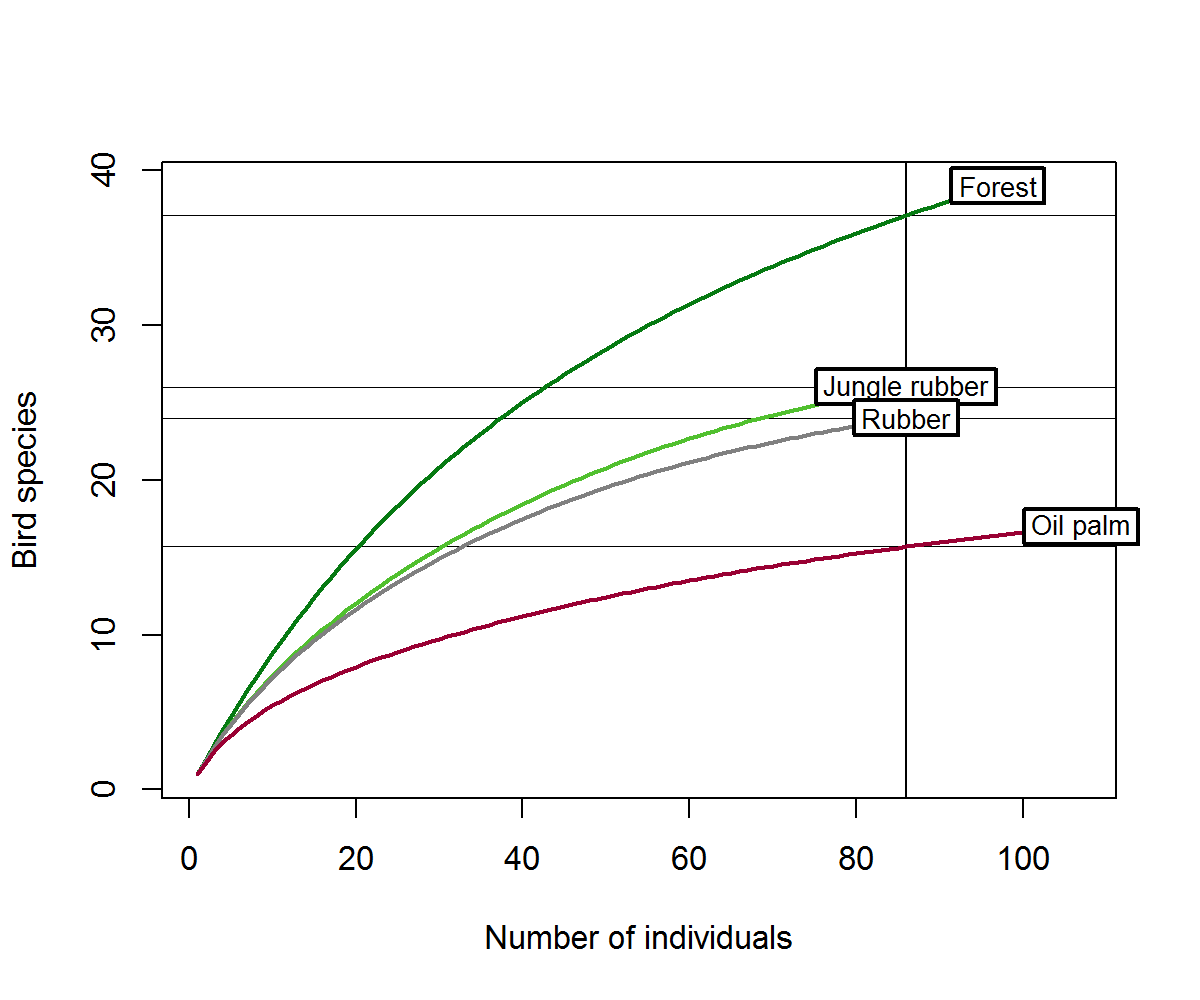

Supplement: S2 Fig — Total species richness was rarefied to the lowest sample size occurring in jungle rubber, where 88 birds were observed. Total rarefied richness forest: 38; jungle rubber: 27; rubber: 25, oil palm: 16. (TIF) [file pone.0154876.s002.tif]

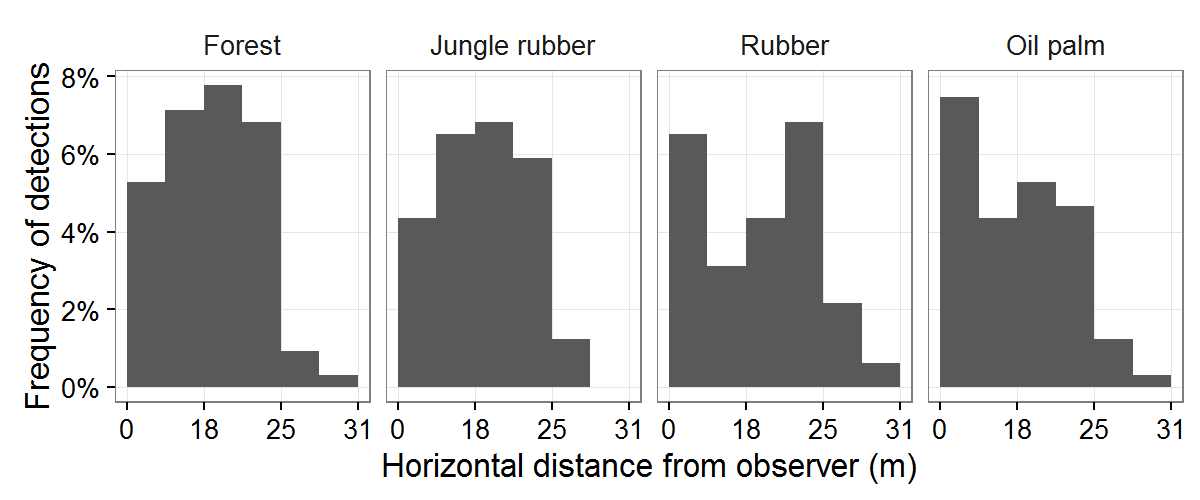

Supplement: S3 Fig — (TIF) [file pone.0154876.s003.tif]

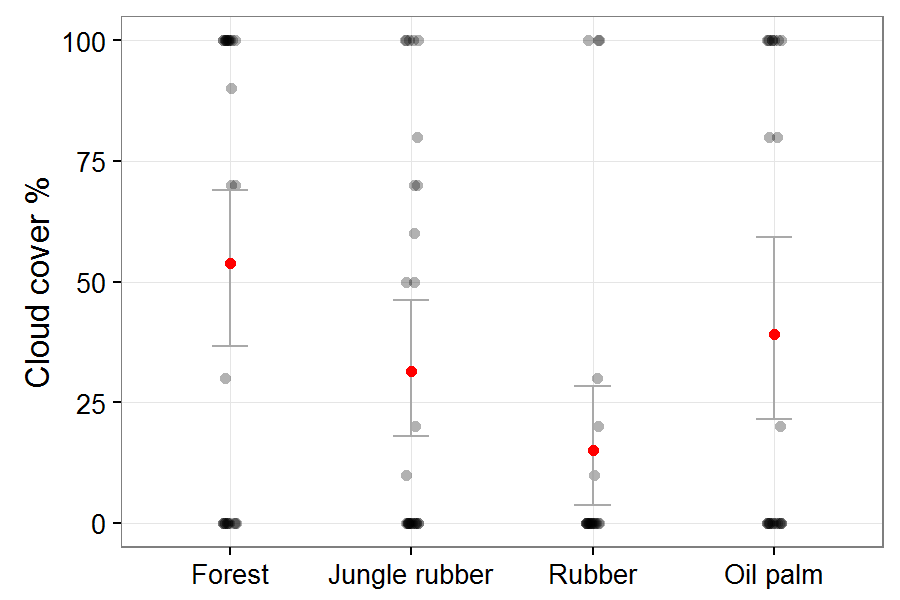

Supplement: S4 Fig — Means are indicated in red, error bars represent the standard error of the mean. (TIF) [file pone.0154876.s004.tif]

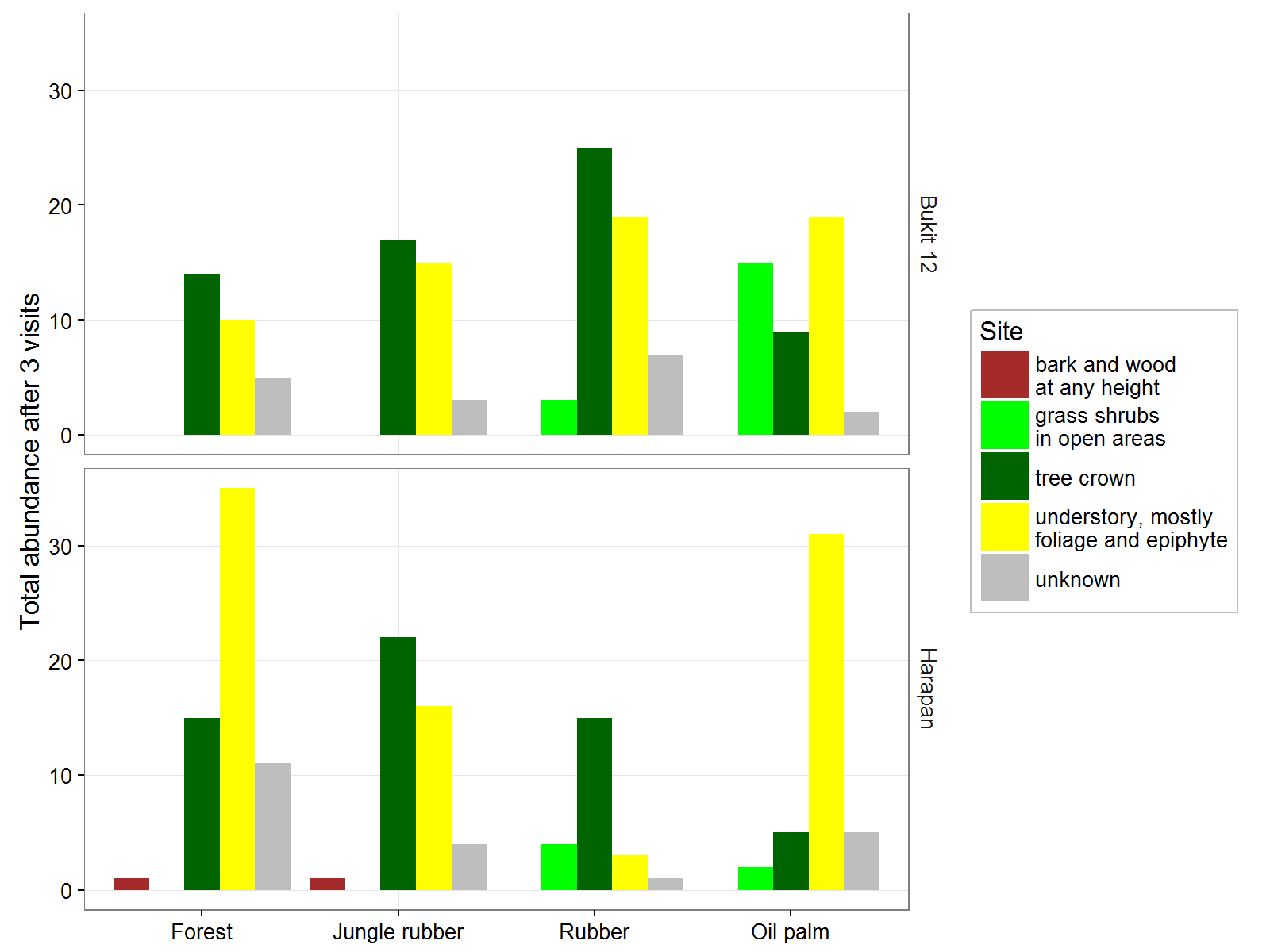

Supplement: S5 Fig — Strata preferences were obtained from Wilman et al. (2014). (TIF) [file pone.0154876.s005.tif]

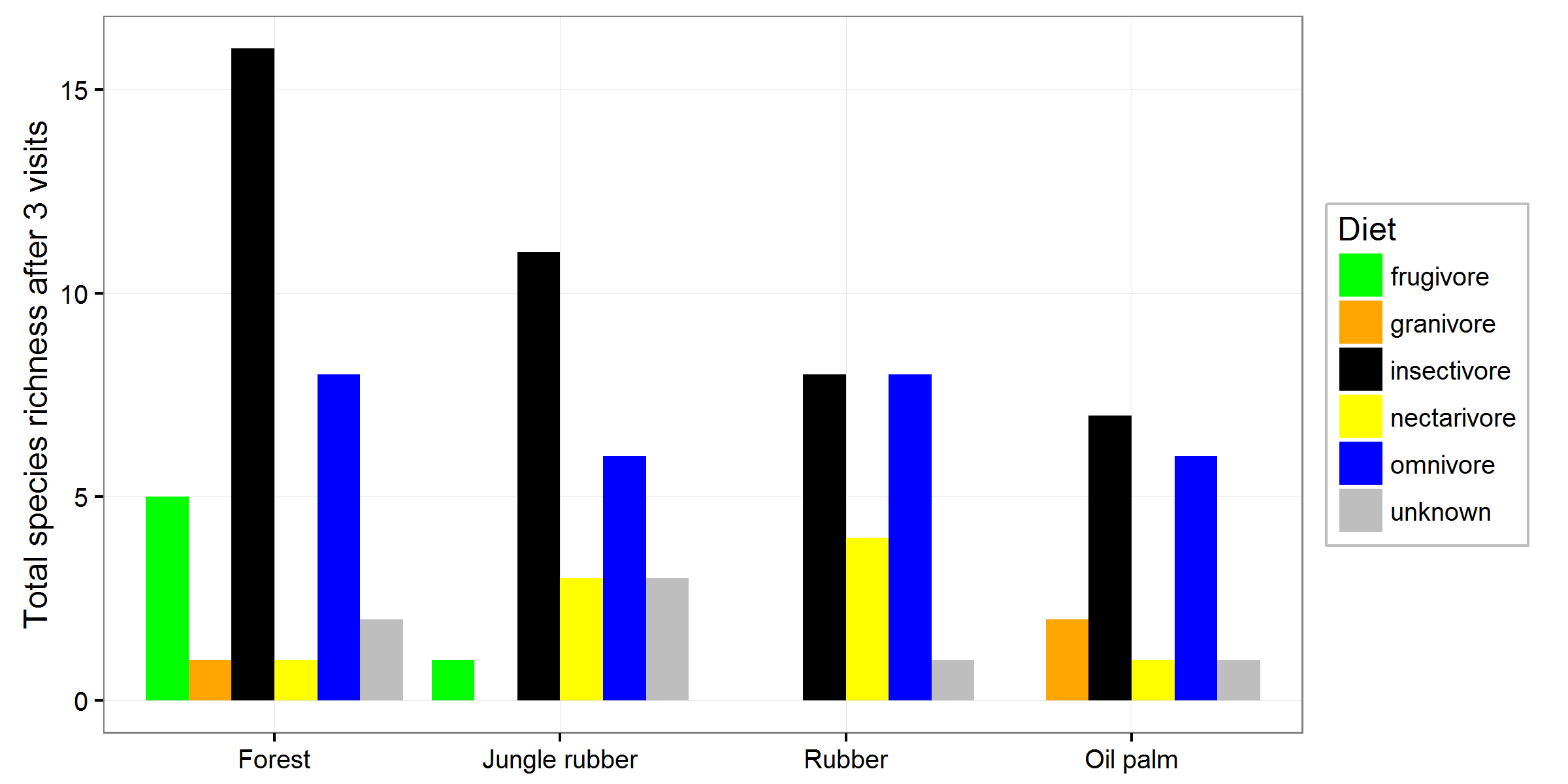

Supplement: S6 Fig — Feeding guild categories were based on Thiollay et al. (1995). (TIF) [file pone.0154876.s006.tif]

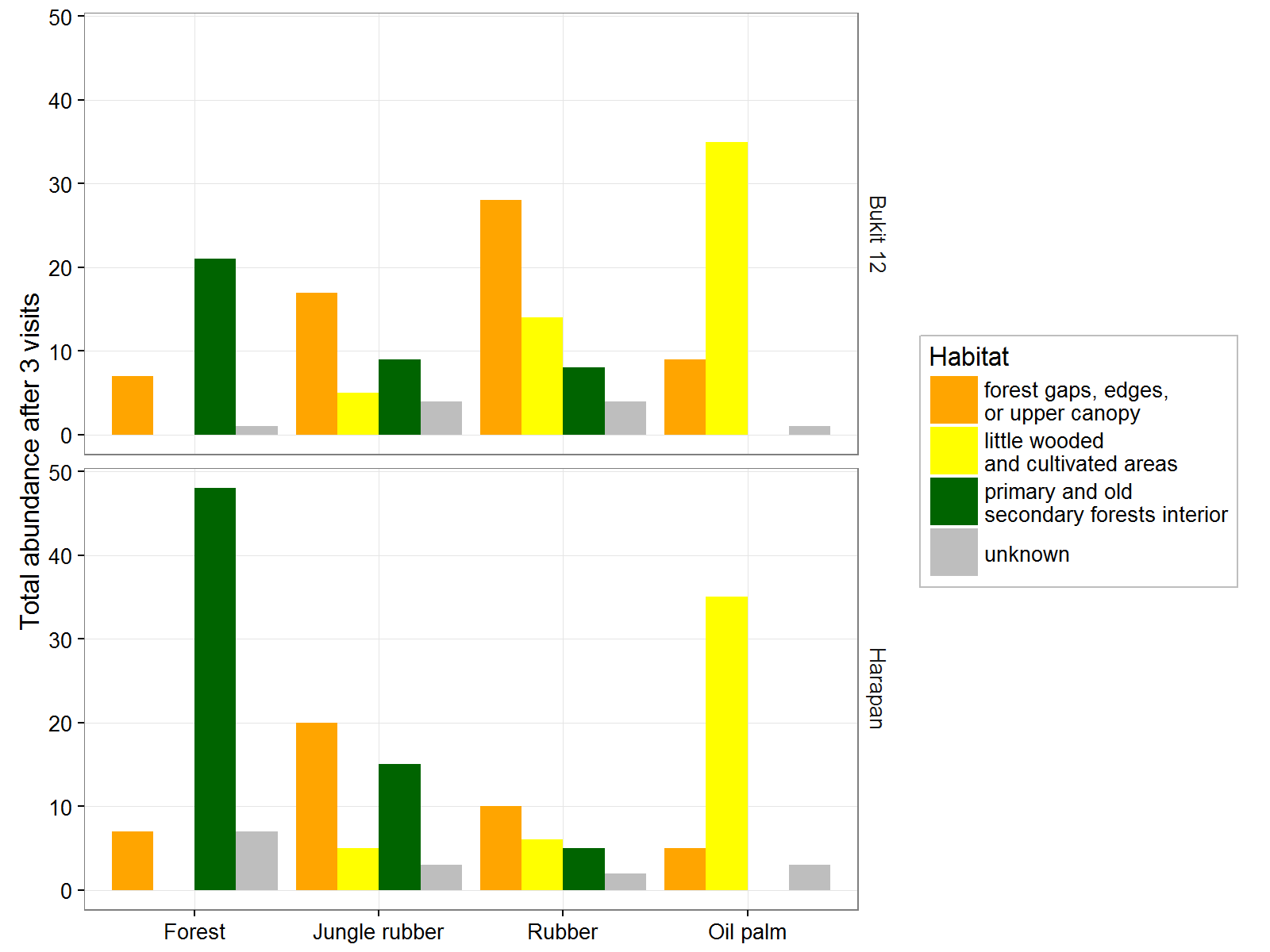

Supplement: S7 Fig — Habitat preferences were mainly obtained from Thiollay et al. (1995). (TIF) [file pone.0154876.s007.tif]
