# Supplementary material for: Bird Responses to Lowland Rainforest Conversion in Sumatran Smallholder Landscapes, Indonesia
Source: PLoS One. 2016 May 25;11(5):e0154876. doi: 10.1371/journal.pone.0154876 (PMC4880215; doi:10.1371/journal.pone.0154876)
Supplement: S1 Questionnaire — (DOCX) [file pone.0154876.s008.docx]

**S1 Questionnaire: Homegarden household questionnaire**

**HOMEGARDENS IN JAMBI, SUMATRA (B09)**

**Date** ___________________ **Location** __________________**_________________________HG** **ID**_________________

**HOUSEHOLD DETAILS**

1. Name of household’s head________________________________________________________________
2. Household address______________________________________________________________________
3. Telephone_____________________________________________________________________________

**SOCIOECONOMIC INFORMATION**

1. What is the main income source of the household? (mark with a **X)**

| Oil palm (owner) |  |
| --- | --- |
| Oil palm (work in company) |  |
| Rubber (owner) |  |
| Rubber (work in company) |  |
| Mining company |  |
| Remittances |  |
| Other |  |
|  |  |

1. How many persons live in the Household? ___________________________________________________
2. Education:

| Children not in age for school |  |
| --- | --- |
| Children in primary (SD) |  |
| Children (SMP) |  |
| Children (SMA) |  |

1. What is the gender of the homegarden responsible (HR)? _________ What is his/her age? ____________

Ethnicity (Mark with an **X**):

| Java |  | Kerinci |  |
| --- | --- | --- | --- |
| Melayu |  | Other |  |
| Batak |  |  |  |
| Batik sembilan |  |  |  |

1. Is the HR a (mark with an **X)**:

| Native from the region |  | Migrant (from other place in Sumatra) |  | Transmigrant (from Java, other place) |  |
| --- | --- | --- | --- | --- | --- |

1. Where does the HR come from? (Name of city or village, and province)___________________________
2. What is the purpose of the homegarden (Mark with an **X**)?

| Self-consumption |  | Selling |  | Both |  |
| --- | --- | --- | --- | --- | --- |

If selling, what is sold?

| Crop name | How much is sold? (kg/week) | Where is sold (exact direction) |
| --- | --- | --- |
|  |  |  |
|  |  |  |
|  |  |  |
|  |  |  |
|  |  |  |

If self-consumption, is it sufficient to meet household needs? (Mark with an **X**)

| Yes |  |
| --- | --- |
| No |  |

**HOMEGARDEN MANAGEMENT**

1. Does the HR use herbicides? (Mark with an **X**)

| Yes |  |
| --- | --- |
| No |  |

If yes, what type of herbicide? (To target weeds)

| Organic | | | Chemical | | |
| --- | --- | --- | --- | --- | --- |
| Name  (or description) | How often?  (last year) | Amount applied  (ml, g) | Name  (or description) | How often?  (last year) | Amount applied  (ml, g) |
|  |  |  |  |  |  |
|  |  |  |  |  |  |
|  |  |  |  |  |  |
|  |  |  |  |  |  |

1. Does the HR do weeding? (Mark with an **X**)

| Yes |  |
| --- | --- |
| No |  |

If yes, how often? (Mark with an **X**)

| Once a year |  |
| --- | --- |
| 2-3 times a year |  |
| More than 3 times a year |  |

1. Does the HR use fertilizer? (Mark with an **X**)

| Yes |  |
| --- | --- |
| No |  |

1. If yes, what type of fertilizer? (To improve nutrients in soil)

| Organic | | | Chemical | | |
| --- | --- | --- | --- | --- | --- |
| Name  (or description) | How often?  (last year) | Amount applied  (ml, g) | Name  (or description) | How often?  (last year) | Amount applied  (ml, g) |
|  |  |  |  |  |  |
|  |  |  |  |  |  |
|  |  |  |  |  |  |
|  |  |  |  |  |  |

1. Does the HR use pesticides?

| Yes |  |
| --- | --- |
| No |  |

If yes, what type of pesticide?

| Organic | | | Chemical | | |
| --- | --- | --- | --- | --- | --- |
| Name  (or description) | How often?  (last year) | Amount applied  (ml, g) | Name  (or description) | How often?  (last year) | Amount applied  (ml, g) |
|  |  |  |  |  |  |
|  |  |  |  |  |  |
|  |  |  |  |  |  |
|  |  |  |  |  |  |

**FURTHER REMARKS**
